# Supplementary material for: The interactome of intact mitochondria by cross-linking mass spectrometry provides evidence for coexisting respiratory supercomplexes
Source: Mol Cell Proteomics. 2017 Dec 8;17(2):216–32. doi: 10.1074/mcp.RA117.000470 (PMC5795388; doi:10.1074/mcp.RA117.000470)
Supplement: Supplemental Data [file supp_RA117.000470_133922_0_supp_23978_fzffwf.zip › spectra_annotation/mito_spectra_annotation/400-1-10-1-11-1.pdf]

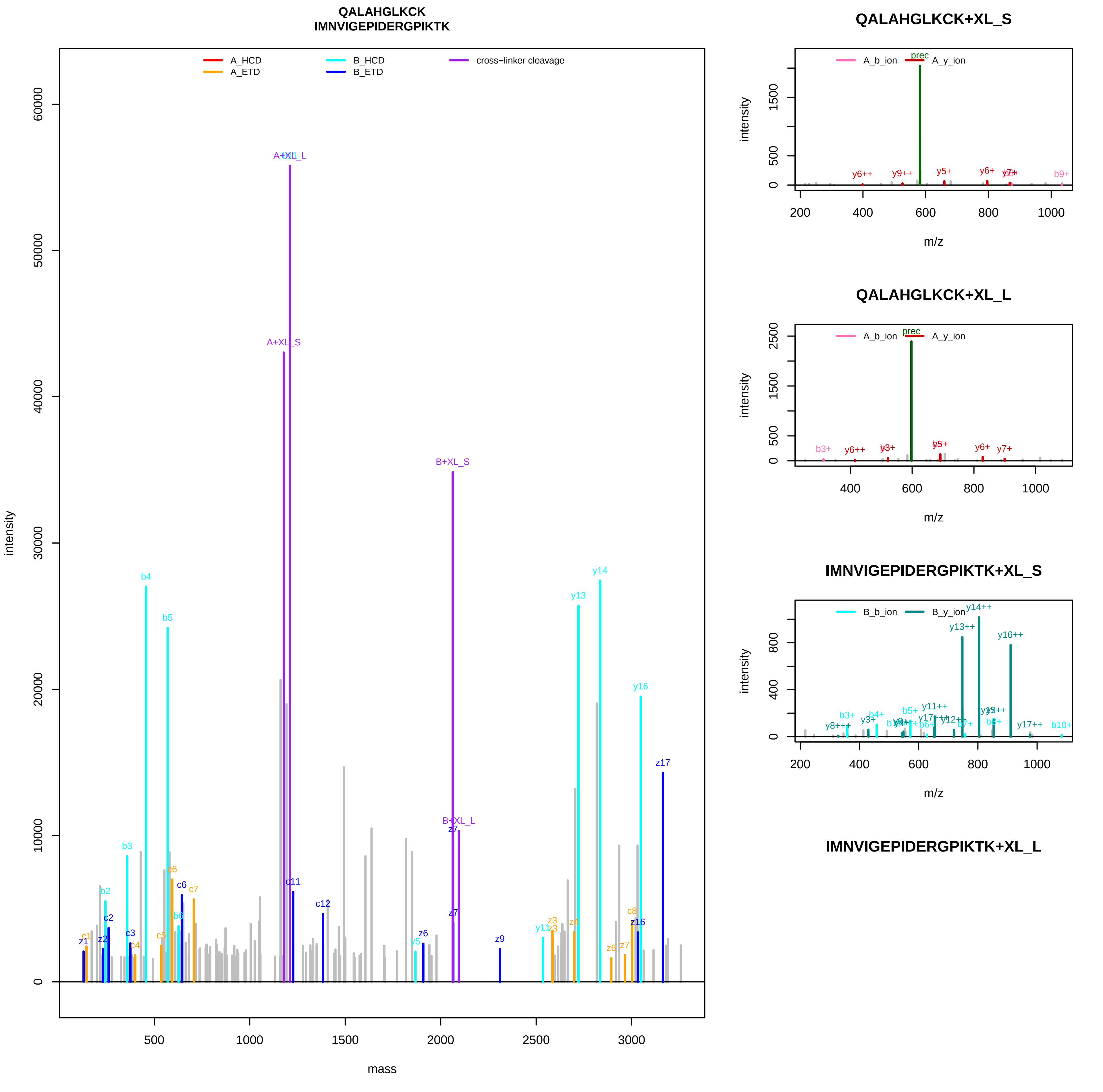
QALAHGLKCK
IMNVIGEPIDERGPIKTK
A_HCD
A_ETD
B_HCD
B_ETD
cross−linker cleavage
intensity
mass
A+XL_L
A+XL_S
B+XL_S
B+XL_L
b2
b3
b4
b5
b6
y5
y11
y13
y14
y16
c1
c2
c3
c4
c5
c6
c7
c8
c11
c12
z1
z2
z3
z4
z6
z7
z9
z16
z17
QALAHGLKCK+XL_S
QALAHGLKCK+XL_L
IMNVIGEPIDERGPIKTK+XL_S
IMNVIGEPIDERGPIKTK+XL_L
A_b_ion
A_y_ion
B_b_ion
B_y_ion
prec
m/z
y6++
y9++
y5+
y6+
y7+
b9+
b3+
y3+
y13++
y14++
y16++
y11++
y8+++
b3+
b4+
b5+
b7+
b8+
y12++
y17++
b10+
